# It might be a dead end: immune checkpoint inhibitor therapy in EGFR-mutated NSCLC

**DOI:** 10.37349/etat.2024.00251

**Published:** 2024-07-19

**Authors:** Ken Akao, Yuko Oya, Takaya Sato, Aki Ikeda, Tomoya Horiguchi, Yasuhiro Goto, Naozumi Hashimoto, Masashi Kondo, Kazuyoshi Imaizumi

**Affiliations:** IRCCS Istituto Romagnolo per lo Studio dei Tumori (IRST) “Dino Amadori”, Italy; Department of Respiratory Medicine, School of medicine, Fujita Health University, Toyoake 470-1192, Japan

**Keywords:** EGFR, immune checkpoint inhibitor, tumor microenvironment, PD-L1

## Abstract

Despite innovative advances in molecular targeted therapy, treatment strategies using immune checkpoint inhibitors (ICIs) for epidermal growth factor receptor (EGFR)-mutant non-small cell lung cancer (NSCLC) have not progressed significantly. Accumulating evidence suggests that ICI chemotherapy is inadequate in this population. Biomarkers of ICI therapy, such as programmed cell death ligand 1 (PD-L1) and tumor-infiltrating lymphocytes (TILs), are not biomarkers in patients with EGFR mutations, and the specificity of the tumor microenvironment has been suggested as the reason for this. Combination therapy with PD-L1 and cytotoxic T-lymphocyte-associated protein 4 (CTLA-4) inhibitors is a concern because of its severe toxicity and limited efficacy. However, early-stage NSCLC may differ from advanced-stage NSCLC. In this review, we comprehensively review the current evidence and summarize the potential of ICI therapy in patients with EGFR mutations after acquiring resistance to treatment with EGFR-tyrosine kinase inhibitors (TKIs) with no *T790M* mutation or whose disease has progressed on osimertinib.

## Introduction

Epidermal growth factor receptor (EGFR) mutations are major genetic variants reported in lung adenocarcinomas, with reported incidences of approximately 50% in Asians and 10–15% in Caucasians [1, 2]. Lung cancer patients with EGFR mutations tend to have little or no smoking history. EGFR-tyrosine kinase inhibitors (TKIs) have been successfully developed for EGFR-mutated non-small cell lung cancer (NSCLC) and continue to stand as robust first-line treatments for advanced NSCLC harboring EGFR mutations [3–6]. Despite their efficacy, resistance to EGFR-TKIs occurs in almost all patients [7]. However, optimal treatment strategies have not yet been established.

For patients who have developed resistance to EGFR-TKIs, treatment strategies based on resistance mechanisms are currently under investigation. However, at present, for patients with any EGFR mutation who have progressed on EGFR-TKIs with no *T790M* mutation or whose disease has progressed on osimertinib, treatment based on the nondriver mutation guideline may be offered [8].

Unlike cytotoxic anticancer drugs, immune checkpoint inhibitor (ICI) therapy is an attractive option due to its potential for a durable response. However, EGFR-mutated NSCLC may be at a disadvantage for immunotherapy because of the lack of smoking history and low tumor mutation burden (TMB). Nevertheless, programmed cell death ligand 1 (PD-L1) expression has been reported to be increased in EGFR-mutant NSCLC [9–11]. Furthermore, in preclinical models, programmed death 1 (PD-1) antibody blockade improved survival in mice with EGFR-driven adenocarcinoma by enhancing effector T cell function and reducing the levels of tumor-promoting cytokines [12], suggesting that ICI treatment for EGFR-mutated cases may be beneficial. Therefore, many clinical trials have been conducted to investigate the efficacy of ICI therapy in patients with NSCLC with EGFR mutations.

However, several clinical trials have shown that the efficacy of ICI therapy in patients with EGFR mutations is limited, and there are concerns regarding its toxicity. Pneumonitis has been reported to be enhanced by the concomitant or sequential use of ICIs [13–15]. Therefore, ICI therapy for patients with EGFR mutations is challenging. Recent data show a trend toward the addition of vascular endothelial growth factor (VEGF) being effective in *EGFR*-mutated cases; however, this is not conclusive.

In addition, ICIs have recently been introduced for locally advanced NSCLC and advanced stages [16–18]. However, whether immune-oncology (IO) should be administered to patients with EGFR mutations at this stage needs to be considered. Thus, summarizing and discussing the findings regarding ICI treatment for patients with EGFR mutations may help us to consider whether patients with EGFR-mutated locally advanced NSCLC should be treated with ICI. In this review, we summarize the treatments, including ICI, and consider whether they are necessary for patients or not.

## Mechanisms and subsequent strategies for EGFR-TKI resistance

Although EGFR-TKIs are the first-line treatment for EGFR-mutated NSCLC, due to their impressive clinical efficacy, almost all patients develop resistance to EGFR-TKIs [7, 19]. Several mechanisms have been reported for the acquired resistance to EGFR-TKIs. One example is the *T790M* second mutation, which is a resistance mechanism against first- and second-generation EGFR-TKIs. *T790M* is reported in approximately 50% of patients who acquire resistance to first-/second-generation EGFR-TKIs [20, 21]. Osimertinib, a third-generation EGFR-TKI developed to overcome this resistance, has shown efficacy against *T790M*-positive *EGFR*-mutated NSCLC and has been approved as a second-line treatment [22]. However, osimertinib was superior to first-generation EGFR-TKIs in the FLAURA trial and has been approved as a first-line therapy in many countries (in certain countries, only as second-line therapy). The mechanisms underlying osimertinib resistance are more diverse than those of the first or second generation [23–25]. Typical examples include *MET* amplification, *C797S* mutations, and signaling pathways other than EGFR, such as *RET* [23, 26]. Many attempts have been made to overcome these mutations as a strategy for osimertinib resistance, including the ORCHARD trial, which used an adaptation strategy for each resistance mutation, and patritumab deruxtecan (HER3-Dxd) and amivantamab plus lasertinib combination therapies, which target broad resistance [27–29]. Prolonged overall survival (OS) has been reported in patients who received platinum-doublet chemotherapy and EGFR-TKIs [30, 31]. As resistance to targeted therapy is expected to develop at a certain point, cytotoxic chemotherapy will continue to hold a prominent position in the treatment of EGFR-mutated NSCLC.

Furthermore, it has been reported that cancers generally become more heterogeneous during the disease [32, 33]. Therefore, the need for broader strategies, such as cytotoxic chemotherapy or immunotherapy, may strengthen after the first and second treatments fail.

## ICI monotherapy for EGFR-mutated NSCLC

The KEYNOTE-001 study was the first attempt to investigate the efficacy of ICIs as a first-line treatment for patients with EGFR-mutated NSCLC. The objective response rate (ORR) to pembrolizumab in four EGFR-TKI-naive patients was 50%, with a median progression free survival (mPFS) of 157.5 days and a median OS (mOS) of 559 days. By contrast, the efficacy was limited in 26 patients previously treated with EGFR-TKIs (ORR, 4%; mPFS, 56 days; mOS, 120 days) [34]. These results led to a phase II trial of pembrolizumab in TKI-naive patients expressing PD-L1. However, the interim analysis did not show the efficacy of pembrolizumab in this population; the ORR in the first 10 patients was 0% and the study was terminated early and considered invalid [35]. CheckMate012 reported that nivolumab monotherapy was less effective in patients with EGFR mutations than those with EGFR wild-type (ORR: 14% vs. 30%; mPFS: 1.8 months vs. 8.8 months; mOS: 18.8 months vs. not reached) [36]. Furthermore, in a meta-analysis of phase II/III trials comparing ICIs (nivolumab, pembrolizumab, and, atezolizumab) with docetaxel in the second-line treatment of NSCLC, the OS of ICIs vs. digital therapeutics (DTX) in patients with EGFR mutations was hazard ratio (HR) 1.11 [95% confidence intervals (CI): 0.80–1.53, *P* = 0.54], indicating that although efficacy has been demonstrated in the overall population, treatment with ICIs is not superior in patients with EGFR mutation-positive [37].

Compared to platinum doublet, in a phase II study (WJOG8515L) comparing carboplatin (CBDCA) plus pemetrexed and nivolumab as second-line therapy after EGFR-TKI failure, nivolumab showed a shorter PFS than CBDCA plus pemetrexed and did not show a survival benefit [38]. In a retrospective analysis (an immunotarget study) investigating the efficacy of ICI monotherapy for each driver gene mutation, the ORR of ICI was 12.0% and the PFS was only 2.1 months (95% CI: 1.8–2.7) in patients with EGFR mutations [39]. The BIRCH and ATRANTIC trials investigated ICI monotherapy in patients with EGFR-mutated PD-L1. In both trials, ICI monotherapy was less effective in EGFR-mutated cases than in wild-type NSCLC [40, 41].

These results indicate that treatment with ICIs is effective in the overall population, but not in patients with EGFR mutations.

The data from these trials (Table 1) suggests that EGFR-mutant NSCLC is less effective than ICI monotherapy. In addition, in a retrospective study, we showed that high PD-L1 expression might not be a predictor of response in patients with EGFR/anaplastic lymphoma kinase (ALK) mutations [42]. A few reports indicate that EGFR is immunologically “cold”, and that the tumor microenvironment (TME) is unfavorable to ICI therapy. Tumors that do not elicit a strong immune response and do not usually respond to immunotherapy are called “cold” tumors. These tumors tend to be surrounded by cells that can suppress the immune response, making it difficult for T cells to attack the tumor cells. Therefore, these TMEs may be responsible for the poor efficacy of ICI monotherapy in EGFR-mutated NSCLC. These trials are summarized in Table 1.

**Table 1:** Immune checkpoint inhibitors monotherapy for EGFR-mutated NSCLC

**Treatment**	**Study name**	**Setting**	**Drugs**	**Phase**	**Efficacy**	**AEs**	**Reference**
IO monotherapy	KEYNOTE-001	Pretreated	Pembrolizumab	I	ORR 50%, mPFS of 157.5 days in four EGFR-TKI-naive patients; ORR 4%, mPFS 56 days in EGFR-TKI treated patients.	No report for EGFR patients.	[34]
	NCT02879994	1st	Pembrolizumab	II	ORR 0%.	TRAE: 46%, no grade 4–5 (38%). 6/7 patients had a TRAE on second-line EGFR-TKI.	[35]
	Checkmate012	2nd	Nivolumab	I	ORR: 14% vs. 30%; mPFS: 1.8 months vs. 8.8 months.	Grade 3–4 in 14 (37%), no G5, in the ITT population.	[36]
	WJOG8515L	2nd	Nivolumab vs. Cb + pemetrexed	II	Nivolumab/Cb + pemetrexed, ORR 9.6% vs. 36.0%, mPFS 1.7 months vs. 5.6 months.	Serious AEs: 25.5% in nivolumab and 16.0% in chemotherapy.	[39]
	BIRCH	1st to 3rd	Atezolizumab	II	ORRs for mutant/wild-type in cohorts 1, 2, and 3 were 23%/19%, 0%/21%, and 7%/18%, respectively.	Grade 3 to 4 AEs occurred in 42% of patients (12% treatment-related).	[40]
	ATRANTIC	Less than 3rd	Durvalumab	II	ORR was 12%.	Treatment-related serious adverse events occurred in 5%.	[41]
Dual-IO	NCT03091491	2nd	Nivolumab vs. nivolumab + ipillimumab	I	ORR 3.2%, PFS 1.22 months in overall cohort.	2/31 of grade 3 TRAE in the overall cohort	[43]
	KEYNOTE 021	Less than 2nd	Pembrolizumab plus ipilimumab	I/II	One of the 12 patients showed an objective response.	-	[44]

IO: immune-oncology; ORR: objective response rate; EGFR: epidermal growth factor receptor; TKIs: tyrosine kinase inhibitors; TRAE: treatment related adverse event; mPFS: median progression free survival; ITT: intent-to-treat; AEs: adverse events; ALK: anaplastic lymphoma kinase. -: no data

## ICIs + EGFR-TKI therapy

The CheckMate012 trial evaluated the combination of nivolumab and erlotinib in 21 patients with EGFR-mutant NSCLC. The PFS of patients previously treated with EGFR-TKIs (*n* = 20) was 5.1 months. Responders were PD-L1 positive or PD-L1 status unknown. No grade 4 or 5 treatment related adverse events (TRAEs) were reported, and 2/21 patients discontinued the study due to toxicity [45]. In contrast, the TATTON trial, which evaluated a combination of osimertinib and durvalumab, raised serious safety concerns. In this trial, 48% of the patients developed at least one grade 3 TRAE, 5/23 developed interstitial lung disease, and all patients discontinued the trial [14]. Furthermore, the CAURAL trial comparing osimertinib with durvalumab as second-line treatment was stopped early due to the early discontinuation of the TATTON trial reported at the same time, and one patient developed pneumonitis [46]. In addition, the combination of cytotoxic T-lymphocyte-associated protein 4 (CTLA-4) and EGFR-TKI for previously treated EGFR-mutant NSCLC was investigated, but most studies were discontinued in the early phase because of low efficacy and toxicity (e.g., tremelimumab and gefitinib [47], ipilimumab and EGFR-TKI or ALK-TKI [44], erlotinib and atezolizumab [48], and pembrolizumab plus gefitinib or erlotinib [49]). These trials are summarized in Table 2.

**Table 2:** Immune checkpoint inhibitors + EGFR-TKI therapy

**Study name**	**Setting**	**Drugs**	**Phase**	**Efficacy**	**AEs**	**References**
CheckMate012	≥ 2nd	Nivolumab and erlotinib	Ib	ORR 15%, DCR 65%, PFS: 5.1 months	G3: 24%, no G4 or G5 TRAEs	[49]
TATTON	≥ 2nd	Durvalumab + osimertinib	Ib	ORR 43%	≥ G3: 48%; ILD occurred in 22% (≥ G3, 8.7%)	[14]
CAURAL	≥ 2nd	Durvalumab + osimertinib	III	ORR 64% in the combination arm	Not sufficient data, grade 2 interstitial lung disease occurred in 1 patient.	[46]
NCT02040064	2nd	Tremelimumab and gefitinib	I	PFS of 2.2 months	G3 TRAE 81%	[50]
NCT01998126	1st	Ipilimumab and erlotinib	I	PFS 27.8 months	Four of the 11 patients had G3 colitis.	[51]
NCT02013219	1st and any	Atezolizumab + erlotinib	Ib	PFS 15.4 months	G3 46%, no G4 or G5 TRAE.	[52]
KEYNOTE 021	1st	Pembrrolizumab + gefitinib (cohort F), Pembrrolizumab + erlotinib (cohort E)	Phase I/II	ORR 41.7% in cohort F and 14.3% in cohort E	G3: 33.3% in cohort F, G3–4: 71.4% in cohort E	[44]

ORR: objective response rate; DCR: dacryocystorhinostomy; PFS: progression free survival; TRAEs: treatment related adverse event; ILD: interstitial lung disease; EGFR: epidermal growth factor receptor; TKI: tyrosine kinase inhibitor

## Dual ICI therapy for EGFR-mutated NSCLC

The KEYNOTE 021 phase I/II study evaluated pembrolizumab plus ipilimumab as second-line or later therapy. In this study, 27% (12/44) of the patients harbored EGFR or ALK, of whom only one showed an objective response [44]. Although several trials have been conducted [43], severe toxicity concerns and limited efficacy were shown.

### ICIs + chemotherapy for EGFR-mutated NSCLC

As a subgroup analysis of patients with EGFR mutations, in the CheckMate012 study evaluating the efficacy of nivolumab in combination with chemotherapy, mPFS and OS were shorter in the EGFR mutation arm than those in the wild-type arm (mPFS: 4.8 months vs. 7.5 months; mOS: 20.5 months vs. 24.5 months) [50]. Additionally, in the IMpower130 trial, CBDCA + nanoparticle albumin-bound paclitaxel (nab-PTX) + atezolizumab did not show superiority compared to chemotherapy in EGFR HR (mPFS: 7.0 months vs. 6.0 months, HR 0.75; 95% CI: 0.36–1.54; mOS: 14.4 months vs. 10.0 months, HR 0.98, 95% CI: 0.41–2.31) [51].

In addition, the CheckMate722 and KEYNOTE789 trials validated chemotherapy + ICI treatment in patients with EGFR mutations. The CheckMate722 trial included 294 patients with EGFR-mutated NSCLC who had progressed with first- or second-generation EGFR-TKIs and did not have the *T790M* mutation and patients who had progressed with osimertinib with or without the *T790M* mutation. Nivolumab and chemotherapy could not show superiority in PFS and OS [52].

The KEYNOTE789 study evaluated CBDCA + pemetrexed + pembrolizumab as a treatment after EGFR-TKI failure in NSCLC harboring EGFR-sensitive mutations (19del or L858R). Similar to the Checkmate722 study, patients who progressed to osimertinib and those who progressed to first- or second-generation EGFR-TKIs without *T790M* mutations were included in this study. The PFS was set to be achieved if HR was 0.70 or less, but resulting in HR 0.80 (95% CI: 0.65–0.97), and OS was set to be achieved if HR was 0.72 or less, but resulting in HR 0.84 (95% CI: 0.69–1.02), exceeding both primary endpoints could not be achieved. Subgroup analysis showed a slightly better OS in the PD-L1-positive group; however, none of the subgroups appeared particularly effective [53].

The ILLUMINATE phase II study evaluated the combination of durvalumab and tremelimumab plus platinum-pemetrexed in EGFR-mutated NSCLC who had experienced disease progression with EGFR-TKIs. The study included *T790M*-negative cohort 1 and *T790M*-positive cohort 2. The ORR was 42% in cohort 1 and 35% in cohort 2, with mPFS of 6.5 months and 4.9 months, respectively, demonstrating modest efficacy for this population. In *T790M*-negative patients, high PD-L1 expression (PD-L1 ≥ 50%) was associated with greater efficacy compared with low expression [54]. These studies are summarized in Table 3.

**Table 3:** Immune checkpoint inhibitors + chemotherapy ± anti-VEGF antibodies for EGFR mutant NSCLC

**Treatment**	**Study name**	**Setting**	**Drugs**	**Phase**	**Efficacy**	**AEs**	**References**
Chemotherapy + IO	CheckMate012	2nd	Nivolumab + platinum doublet	I	EGFR mutated vs. wild type, ORR: 17% vs. 47%, PFS: 4.8 months vs. 7.5 months, OS: 20.5 months vs. 24.5 months.	G3–4: 50%, G5: 0%. (All patients, not only EGFR).	[50]
	IMpower130	2nd	Comparing CBDCA + nab-PTX + atezolizumab with CBDCA + nab-PTX	III	In the subgroup of EGFR/ALK, the mPFS was 7.0 months vs. 6.0 months (HR 0.75, 95% CI: 0.36–1.54).	G3–4: 81% in the combination arm. (All patients, not only EGFR).	[51]
	CheckMate722	2nd	Nivolumab plus chemotherapy vs. chemotherapy	III	PFS: 5.6 months in the nivolumab plus chemotherapy group and 5.4 months in the chemotherapy group.	G3–4: 45% in nivolumab-based therapy and 29% in chemotherapy.	[55]
	KEYNOTE789	2nd	Pembrolizumab plus chemotherapy vs. chemotherapy	III	PFS: 5.6 months in the pembrolizumab plus chemotherapy group and 5.5 months in the chemotherapy group.	G3 ≤ TRAE; 43.7%, irAE 4.5% in combination arm.	[56]
Chemotherapy + dual-IO	ILLUMINATE	2nd	Durvalumab and tremelimumab plus platinum-pemetrexed	II	The ORR was 42% in cohort 1 and 35% in cohort 2, with mPFS of 6.5 months and 4.9 months.	G3–4 colitis 8%, hepatitis 4%, ILD 1%.	[54]
Chemotherapy + IO + anti-VEGF	IMpower150	2nd	Atezolizumab, bevacizumab, carboplatin-paclitaxel (CP). Control arm: BCP, study arm: ACP, ABCP	III	ORR 70.6% for ABCP, 35.6% for ACP, 41.9% for BCP.	G3–4: 64% of ABCP, 68% of ACP, and 64% of BCP.	[57, 58]
Chemotherapy + IO + anti-VEGF	ORIENT	2nd	Scintilimab, IBI305 (bevacizumab biosimilar), pemetrexed + cisplatin (PC). Arm A: SIPC, arm B: SPC, arm C: PC→S	III	Confirmed ORR were 43.9%, 33.1%, and 25.2% in arm A, B, and C, PFS 6.9 months for arm A, 5.5 months for arm B, 4.3 months for arm C.	Grade ≥ 3 treatment-emergent AEs were 54.7% (arm A), 39.3% (arm B), and 51.0% (arm C).	[55]
Chemotherapy + IO + anti-VEGF	IMpower151	2nd	Atezolizumab, bevacizumab, carboplatin-pemetrexed. Control arm: bevacizumab + carboplatin-pemetrexed, study arm: atezolizumab + carboplatin-pemetrexed, atezolizumab + bevacizumab + carboplatin-pemetrexed. Over half of the patients had EGFR/ALK	III	In the subgroup of EGFR/ALK, the mPFS was 8.5 months (95% CI: 6.9–10.3) for atezolizumab + bevacizumab + carboplatin-pemetrexed and 8.3 months (95% CI: 6.9–10.1) for bevacizumab + carboplatin-pemetrexed (HR 0.86, 95% CI: 0.55–1.19).	G3–4: 67.1% of ABCP, G5 5.9% in ABCP.	[59]

irAE: immune-related adverse events; CP: carboplatin-paclitaxel; BCP: bevacizumab carboplatin-paclitaxel; ACP: atezolizumab carboplatin-paclitaxel; PC: pemetrexed + cisplatin; SIPC: scintilimab + IBI305 + pemetrexed + cisplatin; SPC: scintilimab + pemetrexed + cisplatin; EGFR: epidermal growth factor receptor; TRAE: treatment related adverse event; mPFS: median progression free survival; ORR: objective response rate; IO: immune-oncology; OS: overall survival; CBDCA: carboplatin; nab-PTX: nanoparticle albumin-bound paclitaxel; VEGF: vascular endothelial growth factor; NSCLC: non-small cell lung cancer; HR: hazard ratio; CI: confidence intervals; TRAE: treatment related adverse event; ILD: interstitial lung disease; ALK: anaplastic lymphoma kinase. PC→S: pemetrexed + cisplatin → sincilimab

### ICIs + chemotherapy + anti-VEGF antibodies for EGFR-mutated NSCLC

In a subgroup analysis of patients with EGFR mutation in a phase III trial (IMpower150) comparing CBDCA + PTX + bevacizumab + atezolizumab (ABCP) with CBDCA + PTX + bevacizumab (BCP) in the first-line treatment of non-squamous NSCLC, mOS was not reached vs. 18.7 months (HR 0.61, 95% CI: 0.29–1.28) and mPFS was 10.2 months vs. 6.9 months (HR 0.61, 95% CI: 0.36–1.28), showing a trend towards better treatment response in the atezolizumab combination group [60]. Furthermore, OS improvements were sustained with ABCP vs. BCP in sensitizing EGFR mutations in updated analysis (mOS 29.4 months vs. 18.1 months, HR 0.74, 95% CI: 0.38–1.46) [61]. However, this subgroup analysis was not planned in the protocol, and the presence of EGFR mutations was not set as a pre-planned stratification factor, which should be cautioned.

The ORIENT-31 trial is the first prospective phase III trial to show the benefit of anti-PD-1 antibody plus chemotherapy in patients with EGFR-mutated NSCLC who have progressed after treatment with tyrosine kinase inhibitors. In this study, sintilimumab (PD-1 inhibitor) + IBI305 (anti-VEGF) + cisplatin + pemetrexed showed superiority in terms of PFS over chemotherapy and was well tolerated [55]. VEGF is involved in angiogenesis and the formation of a broad immunosuppressive environment. VEGF promotes Treg differentiation and proliferation and inhibits dendritic cell maturation [56]. The combination of PD-1/L1 inhibition and VEGF blockade enhances antigen-specific T-cell migration and modulates the expression of the CD8+ T-cell inhibitory checkpoint in tumors [57, 58, 62]. Therefore, the role of VEGF as an immunomodulator is expected, and elevated VEGF levels have been reported in EGFR-mutant NSCLC [63]. These associations between VEGF and the TME in EGFR-mutated NSCLC support the combined strategy of PD-1 and VEGF inhibition in EGFR cases. However, contradictory results have recently been reported. The IMpower151 trial was presented at the 2023 IASLC World Lung Cancer for the Study of ABCP, or BCP. In the subgroup of patients with EGFR/ALK-positive (*n* = 163), the mPFS was 8.5 months (95% CI: 6.9–10.3) in the ABCP group vs. 8.3 months (95% CI: 6.9–10.1) in the BCP group (non-statistical HR, 0.86; 95% CI: 0.55–1.19) [59]. These results are inconsistent with the improvements in PFS and OS with ABCP observed in the IMpower150 trial. Therefore, the strategy for combination with VEGF blockade remains unknown. However, this is currently the only regimen with promising efficacy.

### ICI therapy for locally advanced NSCLC harboring an EGFR mutation

As mentioned above, the efficacy of ICI treatment in EGFR-mutated cases is limited to advanced stages. However, this does not appear to be the case in early-stage EGFR-mutated NSCLC. The IMpower010 study was a phase III open-label study comparing atezolizumab with placebo after postoperative adjuvant platinum-based chemotherapy for completely resected NSCLC. Disease-free survival (DFS) in stages II–III was significantly longer in the atezolizumab group than in the best supportive care group. In this study, the DFS in the EGFR mutation subgroup in the overall population was HR 0.99 (0.60–1.62), while PD-L1 positive cases showed that the DFS in the EGFR mutation subgroup was HR 0.57 (0.26–1.24), similar to cases without EGFR mutations [17].

In the EGFR mutation subgroup of the KEYNOTE091 trial evaluating postoperative adjuvant pembrolizumab for similar populations, the HR was 0.44 (0.23–0.84), suggesting that it may be better than in non-EGFR mutation cases [64].

KEYNOTE671 is a randomized, double-blind, phase III study that compared pembrolizumab with a placebo after postoperative adjuvant platinum-based chemotherapy for completely resected NSCLC. The primary endpoint, event-free survival, was better in the subgroup of patients with EGFR mutations [HR 0.09 (0.01–0.74)] than in those without EGFR mutations [65].

The AEGEAN study included EGFR mutation cases, but only the results from subjects excluding EGFR mutation cases are available [66].

Thus, unlike patients with advanced disease, patients with EGFR mutations do not seem to be particularly less affected by perioperative treatment than wild-type patients. However, the number of patients with EGFR mutations was small in both studies, which were subgroup analyses. In resectable NSCLC, the levels of CD8+ cytotoxic T cells and CD20+ B cells are associated with OS and DFS, and it has been reported that the higher the number of Tregs, the shorter the OS [67]. In contrast, although PD-L1 expression reflects the presence of tumor-infiltrating lymphocytes (TILs), there are reports that PD-L1 expression is a prognostic marker for resectable NSCLC harboring an EGFR mutation [68]. Therefore, there is no consensus regarding the TME of resectable EGFR-mutated NSCLC. However, osimertinib as a postoperative adjuvant showed a remarkable increase in DFS. Although it is necessary to compare the long-term prognosis, EGFR-TKIs currently have greater benefits as adjuvant treatments. Although immunotherapy for resectable EGFR-mutated NSCLC has DFS benefits, it is less effective than EGFR-TKIs, and there is little need for immunotherapy in clinical practice.

For unresectable stage III NSCLC, durvalumab is the standard treatment after concurrent chemoradiotherapy (CCRT); however, it is reported to be less effective in patients with EGFR [69]. Osimertinib consolidation therapy after CCRT is currently being investigated in the LAURA study.

## Biomarkers and TME of EGFR-mutated NSCLC

Several reports have indicated that the TME has a significant impact on the therapeutic effects of ICIs [70–72].

The most representative biomarker is PD-L1 expression, and it has been reported that PD-L1 expression may be upregulated by multiple pathways in EGFR-mutated NSCLC [9]. However, there are no certain opinions on whether PD-L1 expression is high in EGFR-mutated cases, as a few indicate that PD-L1 expression is high, whereas others indicate that it is low [10, 73]. Real-world studies have reported that PD-L1 expression correlates with the response to first-line osimertinib therapy, and PD-L1 expression is associated with prognosis [74].

No consensus has been observed on the importance of PD-L1 expression in EGFR-mutated NSCLC. However, PD-L1 expression does not appear to be a biomarker for ICI treatment in advanced EGFR cases, and there have been few clinically significant results.

TMB was defined as the number of somatic mutations per megabase in the coding region of a tumor. In advanced NSCLC, there is a significant association between smoking history and genetic alterations and TMB [75], and patients with EGFR-mutated NSCLC are less affected by smoking and therefore have fewer somatic mutations and neoantigens [76]. However, it is reported that TMB is not associated with the therapeutic efficacy of PD-1/L1 blockade in patients with driver mutations [77].

In addition, lymphocyte infiltration into the tumor and surrounding stroma is associated with ICI efficacy, and a higher density of CD8+ TILs is associated with a better ICI response. In contrast, Treg infiltration was associated with poor ICI efficacy. Fewer CD8+ TILs and more Tregs were observed in EGFR-mutated mutations. EGFR-mutated NSCLC is a cold tumor with a non-inflammatory microenvironment. However, the high prevalence of Treg infiltration, which is usually observed in inflammatory microenvironments, is unique. Tregs are induced by EGFR [78].

Various soluble molecules have been reported to interact with EGFR. For example, interleukin-6 (IL6) is reported to be overexpressed in EGFR-mutated mouse models [79], and transforming growth factor-beta (TGF-β) and tumor necrosis factor (TNF) are reported to be increased by EGFR expression [80, 81].

Another potential influence on the TME is the effect of previous EGFR-TKI treatment. Several studies have indicated that EGFR inhibition affects TMEs [73, 78, 79, 82]. Reports have shown that EGFR inhibition improves the TME [78, 82], but the combination of EGFR-TKIs and ICI has shown less clinical benefit in clinical trials.

## Conclusions

Current evidence suggests that ICI therapy for EGFR-positive lung cancer remains inadequate, probably due to the EGFR-specific TME. Various attempts have been made to increase the efficacy of ICI in this population. Combination therapy with CTLA-4 has not shown good results in EGFR cases, whereas the combination of VEGF, chemotherapy, and ICI has shown good results. A better understanding of EGFR-specific TME and consideration of suitable combinations is required to establish treatment strategies, including optimal ICI for this population. However, the evidence currently described is still insufficient for ICI to prolong the prognosis of EGFR-mutated NSCLC, and there is hope for the development of new agents such as ADC drugs and dual antibodies. In addition, adjuvant therapy with PD-L1 inhibitors has been introduced for resectable NSCLC and unresectable stage III NSCLC; however, EGFR-positive NSCLC is unlikely to benefit from ICI, even in these patients, and targeted therapy seems to be the most promising.

## Abbreviations

ABCP: carboplatin + paclitaxel +bevacizumab + atezolizumab
ALK: anaplastic lymphoma kinase
BCP: carboplatin + paclitaxel + bevacizumab
CBDCA: carboplatin
CTLA-4: cytotoxic T-lymphocyte-associated protein 4
DFS: disease-free survival
EGFR: epidermal growth factor receptor
HR: hazard ratio
ICIs: immune checkpoint inhibitors
mPFS: median progression free survival
NSCLC: non-small cell lung cancer
ORR: objective response rate
OS: overall survival
PD-L1: programmed cell death ligand 1
PTX: paclitaxel
TILs: tumor-infiltrating lymphocytes
TKIs: tyrosine kinase inhibitors
TMB: tumor mutation burden
TME: tumor microenvironment
VEGF: vascular endothelial growth factor

## Declarations

### Copyright

© The Author(s) 2024.
